# Investigating pair distribution function use in analysis of nanocrystalline hy­droxy­apatite and carbonate-substituted hy­droxy­apatite

**DOI:** 10.1107/S2053229622003400

**Published:** 2022-04-05

**Authors:** Emily L. Arnold, Dean S. Keeble, J. P. O. Evans, Charlene Greenwood, Keith D. Rogers

**Affiliations:** a Cranfield Forensic Institute, Cranfield University, Shrivenham, Wiltshire, SN6 7LA, United Kingdom; b Diamond Light Source Ltd, Diamond House, Harwell Campus, Didcot, Oxfordshire, OX11 0DE, United Kingdom; c Imaging Science Group, Nottingham Trent University, Rosalind Franklin Building, Nottingham, NG11 8NS, United Kingdom; d School of Chemical and Physical Sciences, Keele University, Keele, Staffordshire, ST5 5BJ, United Kingdom

**Keywords:** powder diffraction, bone mineral, carbonate-substituted hy­droxy­apatite, X-ray diffraction, total scattering, pair distribution function

## Abstract

Hy­droxy­apatite (HA) is a com­plex material, which is often nanocrystalline when found within a biological setting. This work has directly com­pared the structural characteristics derived from data collected using a conventional laboratory-based X-ray diffractometer with those collected from a dedicated pair distribution function (PDF) beamline at Diamond Light Source. In particular, the application of PDF analysis methods to carbonated HA is evaluated. 20 synthetic samples were measured using both X-ray diffraction (XRD) and PDFs. Both Rietveld refinement (of laboratory XRD data) and real-space refinement (of PDF data) were used to analyse all samples. The results of Rietveld and real-space refinements were com­pared to evaluate their application to crystalline and nanocrystalline hy­droxy­apatite. Significant relationships were observed between real-space refinement parameters and increasing carbonate substitution. Understanding the local order of synthetic hy­droxy­apatite can benefit several fields, including both biomedical and clinical settings.

## Abstract 2

Hy­droxy­apatite is a com­plex material, which is often nanocrystalline and substituted within a biological setting. Both long-range and local structures were inter­rogated with X-ray diffraction and X-ray total scattering.

## Introduction

**Figure 1:**
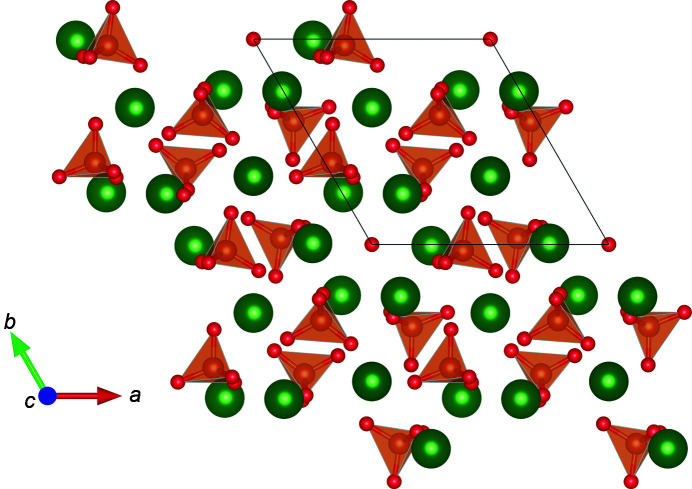
The crystallographic structure for hy­droxy­apatite. Calcium is shown in green, oxygen in red and phospho­rus in orange. Phosphate tetra­hedra are shown in translucent orange.

The structures of biogenically derived apatites are central to biological response in bone and a range of pathological conditions, including cancer (He *et al.*, 2021 ▸). The mineral phase of bone is primarily made up of a calcium phosphate frequently prototyped as hy­droxy­apatite (HA), with a structure shown in Fig. 1 ▸ (Bonucci, 2012 ▸). Many substitutions are commonly present in biogenically derived HA, and several are commonly considered when investigating synthetic HA. The most abundant substitution is carbonate (CO_3_^2−^), which constitutes as much as 5 to 9 wt% of biogenic HA (Von Euw *et al.*, 2019 ▸) and can occupy two distinct locations within the crystal lattice: the *c*-axis channel (substituted for OH^−^ and termed A-type) and the tetra­hedral site (substituted for PO_4_^3−^ and termed B-type) (Rey *et al.*, 2009 ▸). This substitution is known to change with bone disease, such as osteoporosis (Greenwood *et al.*, 2016 ▸; Faibish *et al.*, 2006 ▸; Gadaleta *et al.*, 1996 ▸; McCreadie *et al.*, 2006 ▸), primary and metastatic bone cancers (He *et al.*, 2017 ▸; Zanghellini *et al.*, 2019 ▸), and ageing (Boskey & Imbert, 2017 ▸; Yerramshetty & Akkus, 2013 ▸). Additionally, it has been shown to vary in pathological calcifications with malignancy (Baker *et al.*, 2010 ▸).

Carbonate-substituted HA has the general com­position given below:

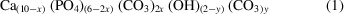




While several different models have been proposed for the precise location of CO_3_^2−^ when substituting for PO_4_^3−^ [usually on one of two faces of the PO_4_^3−^ (Wilson *et al.*, 2004 ▸; Ivanova *et al.*, 2001 ▸) or in the mirror plane positions (Marisa *et al.*, 2016 ▸; Leventouri *et al.*, 2001 ▸) *z* = 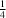 and 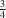 in the space group *P*6_3_/*m*), currently there is little consensus.

Unfortunately, due to the limitations of analytical techniques and the nanocrystalline morphology of biogenic HA, it is difficult to reliably determine carbonate site distribution and concentration. CO_3_^2−^ substitution has been the subject of much research in the past in this context. CO_3_^2−^ substitution has been shown to decrease with increasing tissue age (Paschalis *et al.*, 1996 ▸) and is known to be elevated in osteoporotic tissue (Greenwood *et al.*, 2016 ▸; Faibish *et al.*, 2006 ▸; Gadeleta *et al.*, 2000 ▸; McCreadie *et al.*, 2006 ▸). Increased CO_3_^2−^ substitution is known to increase the solubility of HA (Wopenka & Pasteris, 2005 ▸), although A-type substitution is known to be more thermodynamically stable than B-type (Lafon *et al.*, 2003 ▸). The effects of both A- and B-type CO_3_^2−^ substitution on lattice parameters have been well documented by LeGeros *et al.* (LeGeros *et al.*, 1969 ▸; Zapanta-LeGeros, 1965 ▸) and have been examined further by many studies (Danilchenko *et al.*, 2006 ▸; Madupalli *et al.*, 2017 ▸). However, most studies examine HA synthesized at high tem­per­atures (near 100 °C) to reduce the potential for lattice-bound water. The presence of this lattice-bound water, coupled with additional substitutions common in biogenic HA, further confound the direct inter­pretation of lattice parameters (Brown & Constantz, 1994 ▸; Elliott, 2002 ▸).

In contrast, most studies of biogenic apatites currently rely on Fourier-transform infrared spectroscopy (FT–IR) for carbonate concentration qu­anti­fication and site determination. While total CO_3_^2−^ concentration can be measured relatively accurately, significant difficulty lies in the differentiation of the carbonate site. This is due to the heavy overlapping of the A-type, B-type and labile (or surface) sub-bands of the ν_2_CO_3_^2−^ absorption band (890 to 840 cm^−1^; Rey *et al.*, 1989 ▸; Paschalis *et al.*, 2011 ▸), while the ν_3_CO_3_^2−^ absorption band (1500 to 1395 cm^−1^; Miller *et al.*, 1997 ▸; Rey *et al.*, 1989 ▸) is additionally confounded by several factors, most notably Amide II (1580 to 1480 cm^−1^; Benetti *et al.*, 2014 ▸; Chasov *et al.*, 2018 ▸).

Within the present study, 20 synthetic samples were measured, using conventional laboratory-based X-ray dif­fraction (XRD) and also a dedicated pair distribution function (PDF) beamline at the Diamond Light Source. Samples included highly crystalline and nanocrystalline synthetic HA. This work is the first to com­pare the qu­anti­tative analysis of hy­droxy­apatite using both the Rietveld refinement of laboratory XRD data and the real-space refinement of PDF data. The results of real-space analysis were com­pared to CO_3_^2−^ concentration for all samples to determine systematic structural differences correlated to CO_3_^2−^ increases. This work uses the largest number of HA samples analysed using real-space refinement and includes a range of crystallinity and CO_3_^2−^ concentrations. We hypothesize that highly crystalline samples will result in a crystallographic structure close to that of stoichiometric HA, while nanocrystalline samples will be characterized by more variation with both local and average structure refinement. It is expected that the examination of local order of carbonated HA will be beneficial to a range of disciplines, including clinical, biomedical and forensic settings.

## Materials and methods

### Materials

A range of synthetic apatites were synthesized as described previously (Arnold *et al.*, 2020 ▸). Furthermore, the samples formed three groups which are summarized in Table 1 ▸: (*a*) two National Institute of Standards and Technology Standard Reference Materials (NIST SRM), (*b*) five carbonated HA samples obtained from the University of Exeter and (*c*) 13 synthetic HA samples synthesized using methods based on Jarcho *et al.* (1976 ▸) and Merry *et al.* (1998 ▸) for stoichiometric and carbonated HA, respectively. Subsequently, all samples synthesized at high-tem­per­ature (group *b*) may be referred to as *crystalline* and all samples synthesized at low tem­per­ature (group *c*) may be referred to as *nanocrystalline*. All samples were ground manually using an agate mortar and pestle. The resultant powders were sieved using a 106 µm mesh to reduce preferred-orientation effects and to improve particle statistics. In addition to laboratory XRD and attenuated total reflectance (ATR) FT–IR, X-ray total scattering techniques and real-space refinement were used to characterize all samples.

### ATR–FT–IR

ATR–FT–IR was used to estimate the carbonate levels in all the samples. All data were collected using a Bruker Alpha instrument with a diamond ATR crystal, and a scan range from 4000 to 400 cm^−1^, with 64 averaged scans and a 4 cm^−1^ resolution. Spectra were analysed using *Spectrum* (Perkin­Elmer); an example of this analysis can be seen in Fig. S1 in the supporting information. The net area was measured for the ν_1_ν_3_PO_4_^3−^ and ν_2_CO_3_^2−^ bands. Each spectrum was deconvoluted until peak centres were apparent near 878, 873 and 866 cm^−1^ (Rey *et al.*, 1989 ▸). *PeakFit4* (Sigmaplot) was used to fit three peaks to all samples with a measurable carbonate content after the spectra were limited to 910 to 840 cm^−1^ and the baseline corrected. The peak centres were fixed at values determined with deconvolution. A least-squares fitting technique was used to fit three Voigt peaks to each spectrum. The peak areas from all samples from groups synthesized at high tem­per­atures were used to create a CO_3_^2−^ concentration calibration curve, using the ratio of ν_2_CO_3_^2−^:ν_1_ν_3_PO_4_^3−^ peak areas.

### XRD

All laboratory-based XRD data was collected using a PANalytical X’Pert Pro Multi-Purpose Diffractometer with a Cu radiation source in Bragg–Brentano geometry. A PIXcel strip detector was used to collect data in the range 10–80° 2θ. *Topas-ACADEMIC* (Version 6; Coelho, 2018 ▸; Coelho *et al.*, 2015 ▸) was used to perform Rietveld fits for all samples, using the *P*1 structure detailed below. Temperature factors were fixed and lattice parameters (LPs) were calculated from all samples after spiking with an inter­nal silicon standard (NIST SRM 640c) using a full-profile refinement procedure. Coherence length (CL) was determined from 002 and 030 Bragg maxima using Scherrer’s equation (Venkateswarlu *et al.*, 2010 ▸), from a split pseudo-Voigt peak shape.

### Total scattering

Experimental PDFs were acquired from the I15-1 beamline at Diamond Light Source, using 76.7 keV radiation (λ = 0.161669 Å). Total scattering data was collected using a PerkinElmer XRD4343 detector positioned 234.09 mm from the sample. DAWN (Filik *et al.*, 2017 ▸) was used to integrate the two-dimensional (2D) area data to one-dimensional (1D) line data. PDFs were processed using *GudrunX* (Soper & Barney, 2011 ▸), with *Q*_min_ = 0.5 Å^−1^ and *Q*_max_ = 25.6 Å^−1^. The com­position was calculated using the CO_3_^2−^ concentration for all samples [assuming no additional substitution, Equation (1) was charge balanced to determine the HA com­position]. Den­sity was calculated from HA com­position and lattice parameters were determined by XRD.

Analysis of the PDF data was com­pleted using *Topas-ACADEMIC* (Version 6; Coelho, 2018 ▸; Coelho *et al.*, 2015 ▸) in the *r* range 1–50 Å. Instrumental damping was determined by refinement of a silicon standard (NIST SRM 640c). Due to the typical platy morphology of nanocrystalline HA, a nanosheet damping profile was applied to the model, with refinement of the nanosheet thickness, *t*, according to Kodama *et al.* (2006 ▸) and given below:

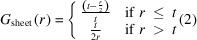

The same *P*1 structure used for XRD analysis was also employed for PDF refinement, detailed below. To model correlated motion at a low radius, a spherical function was applied to determine the tem­per­ature factor (Chater, 2017 ▸). Each spherical function is dependent on three parameters: *beqlo*, *beqhi* and the *beq* radius. The effective tem­per­ature factor (*B*) is *beqlo* at *r* = 0 and *beqhi* at *r* = 2*radius (see Fig. S2 in the supporting information).

### *P*1 structure

**Figure 2:**
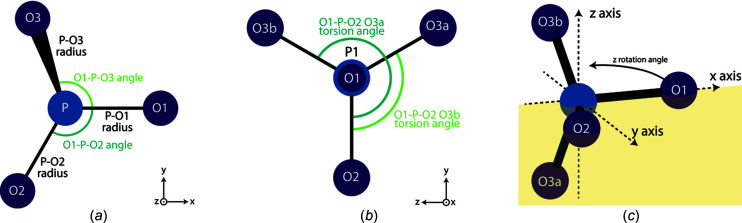
Parameters used to generate rigid bodies, showing (*a*) the bond lengths and O1—P—O2 and O1—P—O3 angles, (*b*) the torque angles O3a and O3b with the *xy* plane (bis­ecting atoms P, O1 and O2) and (*c*) rotation about the *z* axis, where atoms P, O1 and O2 are now positioned on the mirror plane.

A *P*1 structure based on the structure from Kay *et al.* (1964 ▸) was used for real-space and Rietveld refinements, with symmetry constraints manually imposed during fitting. This *P*1 structure was chosen to eliminate false peaks within the calculated PDF, which arose from multiple sites within the usual *P*6_3_/*m* space group that are partially occupied. The assumption that these sites are all simultaneously partially occupied produces the false peaks, as some combination of sites present an unphysical structure. Each PO_4_^3−^ anion was modelled as a rigid body, with five atoms: P, O1, O2, O3a and O3b. All rigid-body parameters are described in Fig. 2 ▸. An example of this *P*1 structure is available as a .CIF in the supporting information.

### Statistics

Linear regressions were performed in *Minitab17* (https://www.minitab.com) to determine correlation between parameters.

## Results and discussion

### Comparison of XRD and PDF methods

To evaluate the utility of PDF methods and real-space refinement for HA, the results of the Rietveld refinement of Bragg data and the real-space refinement of PDF data were com­pared. In addition to this, the coherence length was calculated using Scherrer’s equation (Venkateswarlu *et al.*, 2010 ▸). Exemplary Rietveld and real-space refinements for three samples (SRM 2910b, 1.24 wt% and 7.98 wt%) alongside FT–IR spectra are shown in Fig. S3 in the supporting information.

**Figure 3:**
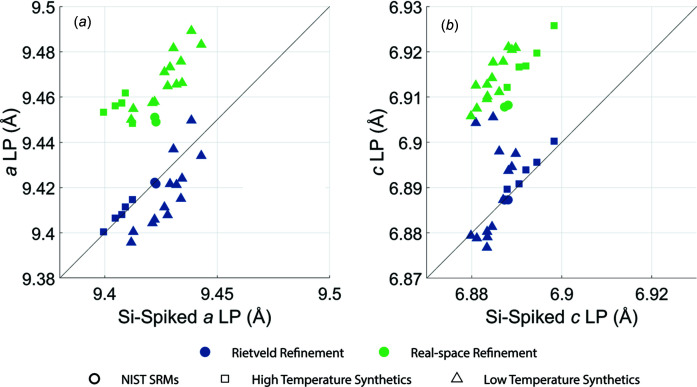
Comparison of the lattice parameters calculated from both the Rietveld refinement of Bragg data and the real-space refinement of PDF data against those calculated from inter­nally Si-spiked samples.

The Rietveld fit of Bragg data gives a more accurate calculation of LPs (com­pared to an Si-spiked sample) (Fig. 3 ▸). Clear differences in accuracy are seen between nanocrystalline and crystalline samples. For both the *a* and the *c* axes, relatively crystalline samples give accurate LP refinements. However, nanocrystalline samples show a systematic underestimation for the *a* axis, and an increased variance for the *c* axis. In contrast, while PDF refinement gives a systematic overestimation of LPs, this overestimation is consistent for all samples, both crystalline and nanocrystalline. Additionally, a slight increase is seen in NIST SRM 640c when real-space refinement is com­pared to the certified value. All experimental values for NIST SRM 640c and 2910b, as well as the certified values, are given in Table 2 ▸.

Linear regressions were performed between equivalent parameters within the Rietveld and real-space refinements, with the results shown in Table 3 ▸. Inter­estingly, few parameters have a correlation coefficient above 0.5, notably both the *a* and *c* LPs (0.76 and 0.59, respectively) and the occupancy of P (0.69). Some Rietveld refinements of Bragg data have consistently given relatively unphysical results, particularly for the P—O bond length, which is often either unreasonably low (around 1.4 Å) or high (around 1.7 Å). Similarly, the O—P—O angles used for rigid-body construction are consistently higher or lower than the ideal value of 109.5° (130 and 105°, respectively).

Consideration of the atomic fractional coordinates shows a much larger range of values from Rietveld refinement than those resulting from PDF refinement, shown in Fig. S4 of the supporting information. Similar trends can be seen for the rigid-body parameters as well. It should be noted that where there is a large range of values refined by a Rietveld method, samples which are particularly crystalline are refined to values which are more similar to previously reported values (Kay *et al.*, 1964 ▸; Shamrai *et al.*, 2014 ▸; Morgan *et al.*, 2000 ▸; Ivankovic *et al.*, 2009 ▸).

When considering rigid-body parameters, shown in Fig. S5 in the supporting information, several notable features from the Rietveld refinements of Bragg data are immediately apparent, as mentioned previously. P—O bond lengths are either smaller (for O1 and O2) or much larger (for O3) than the ideal value of 1.52 Å and even outside the range of values (1.45–1.60 Å) frequently reported in the literature (Shamrai *et al.*, 2014 ▸; Mir *et al.*, 2012 ▸; Morgan *et al.*, 2000 ▸; Guo *et al.*, 2003 ▸; Wilson *et al.*, 1999 ▸; Kay *et al.*, 1964 ▸). Similarly, the angles are largely different from the ideal value of 109.5°, and even sometimes far removed from the 105–112° range commonly seen when refining crystalline HA (Kay *et al.*, 1964 ▸) and provided within the Protein Data Bank (PDB; https://www.rcsb.org/) (Feng *et al.*, 2004 ▸). Further com­parison of the torsion angles between the plane containing O1—P—O2 and O3 shows that Rietveld refinements vary substanti­ally from both the ideal (120 and 240°) and the experimental (116.7 and 236.3°). In contrast, real-space refinements of PDF data give torsion angles closer to the experimental values (around 234 and 115°).

Most of the refined occupancy values are significantly different when com­paring the results of Rietveld refinements and PDF refinements (illustrated in Fig. S6 of the supporting information). For most occupancy values, Rietveld refinement values tend to give greater occupancies for nanocrystalline samples when com­pared to real-space refinement values. The occupancy of phospho­rus is exceptional, with a significant correlation of 0.69 (*p* < 0.001).

**Figure 4:**
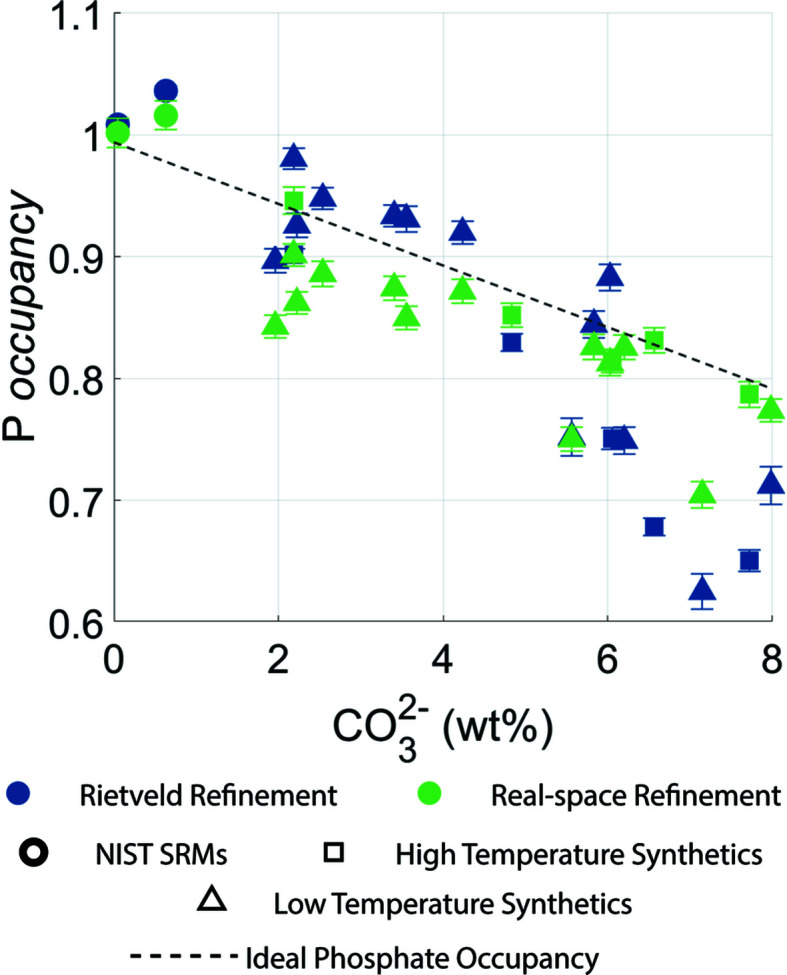
Phosphate occupancy and CO_3_^2−^ concentration for both the Rietveld refinement of Bragg data and the real-space refinement of PDF data. Error bars represent estimated standard deviations.

The expected P-atom occupancy was calculated from the experimentally determined CO_3_^2−^ concentration, shown in Fig. 4 ▸. Linear regressions were performed between the ex­pected P-atom occupancy and the experimental P-atom occupancy for both the Rietveld and the real-space refinement; the results are presented in Table S4 of the supporting information. While both relationships are significant, real-space refinement gives a stronger relationship (*R* ^2^ = 0.72 for real-space refinement as opposed to *R* ^2^ = 0.62 for Rietveld refinement), which is also closer to the expected value and is apparent from the correlation coefficient being closer to 1.

**Figure 5:**
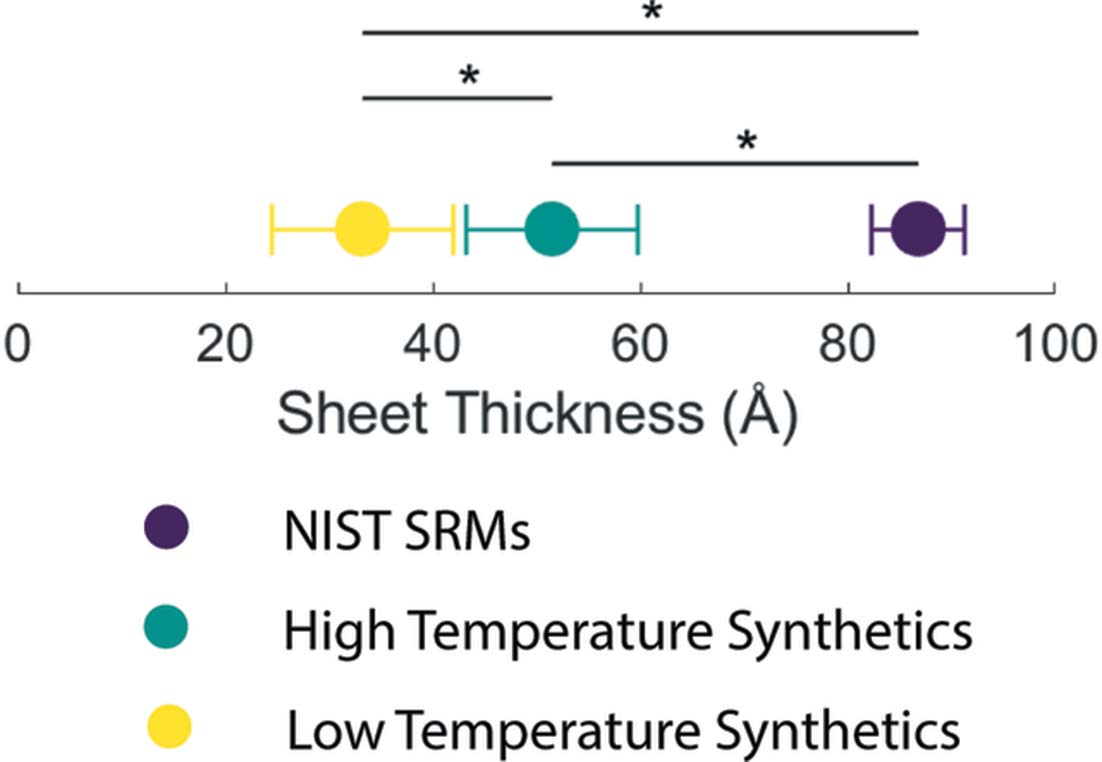
The mean and standard deviation of sheet thickness (Å) for three groups of samples. Significant differences are shown with an asterisk (*) (*p* < 0.05 for all).

Sheet thickness values, determined from real-space refinement of PDF data, are presented in Fig. 5 ▸. Significant differences are seen between the low- and high-tem­per­ature carbonated samples (*p* = 0.003). When sheet thickness was com­pared to coherence length (CL) of the 002 and 030 Bragg reflections (Fig. S7 in the supporting information), significant and strong relationships between sheet thickness and 002 CL and 030 CL (*p* < 0.001 and *R^2^* > 0.8 for all) was demonstrated. However, it should be noted that all CLs determined from long-range, rather than local, analysis see nearly a tenfold increase com­pared to the same sample when analysed using real-space refinement.

#### Discussion

In general, parameters refined using Rietveld and real-space refinements were similar for the most crystalline materials. Furthermore, most parameters refined over a larger range of values with Rietveld refinement when com­pared to real-space refinement of PDF data. For some of these parameters, particularly rigid-body parameters, the resulting structure is not physically plausible, particularly for nanocrystalline samples. The increase seen in LPs when calculated from the PDF could also potentially arise from amorphous content within the material, as may affect calculation of LPs from the PDF, and amorphous calcium phosphate (ACP) has been reported within these materials in the past (Posner & Betts, 1975 ▸; Betts *et al.*, 1975 ▸). While this study refined the PDF in the range 1–50 Å (ACP has been shown to have local order near 10 Å; Betts *et al.*, 1975 ▸), it is possible that any amorphous material present would still affect the calculations of LPs. Due to these apparent differences in Rietveld and real-space refinement results, particularly for nanocrystalline samples, we propose that real-space analysis of PDF data is better suited to the accurate analysis of the structure of nanocrystalline HA.

For platy nanocrystalline samples, sheet thickness typically ranged from ∼20 to 40 Å, consistent with values seen in the literature (Hu *et al.*, 2010 ▸; Kim *et al.*, 1995 ▸; Von Euw *et al.*, 2019 ▸). Due to the symmetry of HA, if a platy crystallite is assumed, the 030 CL will be com­prised of a convolution of the shortest two axes of the plate [commonly reported to be ∼15–30 and ∼50–700 Å, respectively (Hu *et al.*, 2010 ▸; Kim *et al.*, 1995 ▸; Von Euw *et al.*, 2019 ▸)]. For CL calculation from Bragg data (and thus long-range crystal structure), there is little insight available for the smallest dimension of the platy nanocrystalline HA. Inter­estingly, the strong correlation between sheet thickness and both 002 and 030 CL indicates that crystals may experience proportional growth in each axis. While real-space analysis presents a technique to inter­pret the coherence of the smallest axis of these platy crystallites, to evaluate the accuracy of this method, samples would need to be characterized by a method which allows direct measurement of the anisotropic crystallite size. Limitations are still present here, as the calculated *G*(*r*) and the damping profile are both isotropic (Usher *et al.*, 2018 ▸). Care should be taken with hy­droxy­apatite, particularly at low tem­per­atures, due to the heavily anisotropic nature of this material.

### Application of PDF methods to com­positional analysis

To evaluate the application of total scattering and real-space refinement to carbonated HA, the results of the real-space analysis were com­pared to CO_3_^2−^ concentration for all the samples examined.

Linear regression of atomic fractional coordinates, rigid-body parameters, site occupancy and CO_3_^2−^ concentration were calculated to determine significant relationships, which are summarized in Table 4 ▸. It should be noted that the lack of significance in the majority of the high-tem­per­ature samples is likely due to limited sample numbers (*N* = 5) when com­pared to low-tem­per­ature samples (*N* = 13). When examining the high-tem­per­ature synthetics, even some regressions which show relatively high *R* ^2^ values (*R* ^2^ > 0.75) are not significant.

Plots of the fractional coordinates with respect to CO_3_^2−^ concentration are given in Fig. S8 of the supporting information. Ca1 *z* is positively correlated for both high- and low-tem­per­ature synthetics (*p* = 0.001 and 0.004, respectively). This causes a reduction in the separation between the mirror-related calcium ions at the same *x*,*y* fractional coordinates, *e.g. x* = 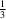 and *y* = 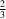 or *x* = 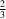 and *y* = 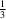 , to move towards each other (towards *z* = 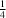 and *z* = 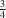 , respectively). The value of the *z* coordinate of the O(H) atom is negatively correlated for both high- and low-tem­per­ature synthetics (*p* = 0.003 and 0.001, respectively). This causes both O atoms in the ion channel to move towards the origin.

When examining rigid-body parameters, shown in Fig. S9 of the supporting information, there are none which are significantly correlated to CO_3_^2−^ concentration for the high-tem­per­ature synthetics. However, for the low-tem­per­ature synthetics, the bond length between atoms P and O3 is negatively correlated to CO_3_^2−^ concentration. Furthermore, the O1—P—O2 and O1—P—O3 angles are also significantly positively and negatively correlated to CO_3_^2−^ concentration, respectively (*p* = 0.002 and 0.001, respectively).

When *beqlo* parameters were com­pared to CO_3_^2−^ concentration, no significant correlations were noted (see Fig. S10 in the supporting information). In contrast, when *beqhi* parameters were com­pared, all but *beqhi* O2 and O(H) showed strong positive relationships (also seen in Fig. S10).

**Figure 6:**
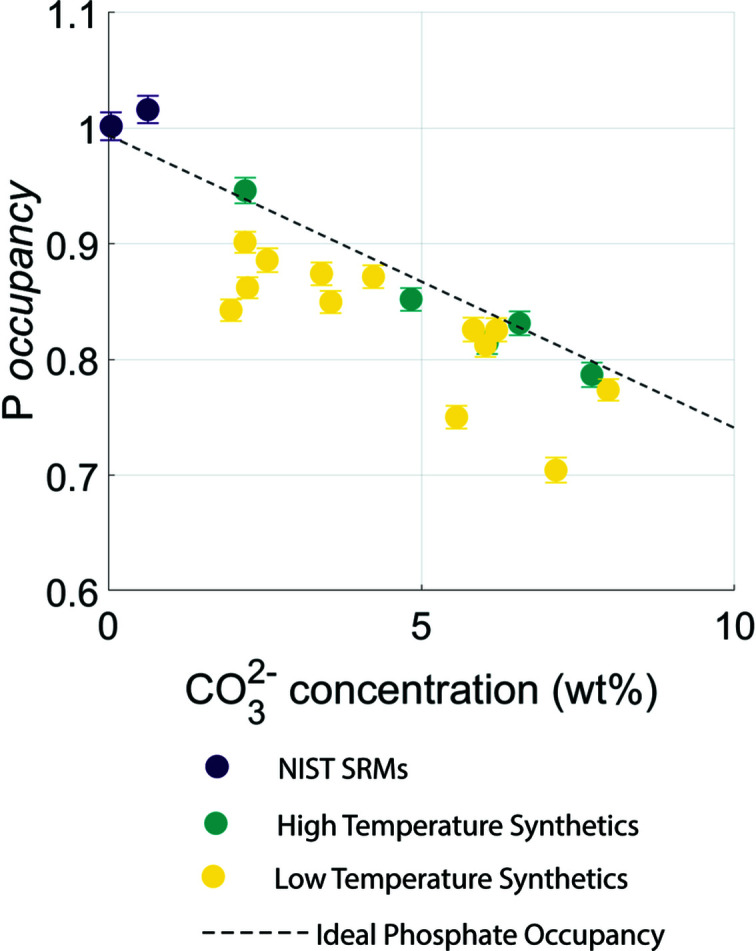
The occupancy of phospho­rus plotted against CO_3_^2−^ concentration, com­pared to ideal P-atom occupancy. Error bars represent estimated standard deviations.

Relationships between site occupancy and CO_3_^2−^ concentration are given in Fig. S11 (see supporting information). Of particular inter­est here is the occupancy of the phospho­rus ion. For both high- and low-tem­per­ature synthetic samples, there is a significant negative relationship with total CO_3_^2−^ concentration (*p* = 0.004 and 0.001, respectively). While the regression lines for these two relationships are slightly different (low-tem­per­ature synthetics tend to have a slightly lower occupancy than high-tem­per­ature synthetics), they are both remarkably close to each other and an ideal model (see Fig. 6 ▸). Here the ideal model is calculated using Equation (1), assuming a vacancy within the phospho­rus site.

Aside from the occupancy of phospho­rus, the occupancy of atom O2 is the only other parameter which is significantly correlated to CO_3_^2−^ among samples synthesized at both high and low tem­per­ature (*p* = 0.035 and 0.001, respectively), both with a negative correlation. In samples synthesized at low tem­per­ature, the occupancy of atom O3 is significantly (positively) correlated to the total CO_3_^2−^ concentration (*p* = 0.001).

While the occupancy of Ca1 in the low-tem­per­ature synthetics is significantly correlated to CO_3_^2−^ concentration (*p* = 0.004), the occupancies of both Ca1 and Ca2 decrease with increasing CO_3_^2−^ concentration. This is consistent with what would be expected with increasing CO_3_^2−^ substitution, as Ca vacancy (or indeed Na^+^ substitution) is used as a charge-balancing mechanism.

The occupancy of O(H) has a significant positive correlation in the low-tem­per­ature samples and seems to be positively correlated to the O—H bending and stretching vibration bands of H_2_O (apparent in the FT–IR spectra at 1650 and 3450 cm^−1^, respectively) (Shi *et al.*, 2005 ▸; LeGeros *et al.*, 1978 ▸; Rey *et al.*, 1995 ▸).

#### Discussion

With the presence of several significant relationships between CO_3_^2−^ concentration and fractional coordinates, rigid-body parameters and site occupancies [*e.g.* Ca1 *z*, O(H) *z* and P-atom occupancy], it is apparent that real-space refinements have the potential to be used for CO_3_^2−^ site determination. For samples synthesized at high tem­per­atures, the occupancy of phospho­rus is very close to a stoichiometric model. While samples synthesized at low tem­per­atures show a little more variation from the ideal model, this would potentially be expected due to other imperfections within the crystallite (*e.g.* vacancies and substitutions), which is expected in intentionally carbonated samples due to the synthesis procedure (Hing *et al.*, 1999 ▸).

While there is no significant correlation between CO_3_^2−^ concentration and the occupancy of atom O1, there is a negative correlation between CO_3_^2−^ concentration and atom O2 in both low- and high-tem­per­ature synthetics, and a positive correlation with O3 in the low-tem­per­ature synthetics. The lack of relationship between CO_3_^2−^ concentration and O1 occupancy may indicate that this position is occupied during CO_3_^2−^ substitution. Thus, the significant decrease in occupancy of the O2 atom could be caused by preferential substitution of CO_3_^2−^ in the C1 position over the C2 position, as described by Ivanova *et al.* (2001 ▸).

## Conclusions

Apatite possesses a somewhat enigmatic structure that continues to challenge analytical characterizations. In our study, significant changes are seen in the local order of carbonated and stoichiometric HA, as would be expected from previous studies of the local and average order of synthetic hy­droxy­apatites (Arnold *et al.*, 2020 ▸; Marisa *et al.*, 2016 ▸; Ivanova *et al.*, 2001 ▸; Fleet & Liu, 2005 ▸, 2004 ▸). The use of real-space refinement in com­parison to Rietveld refinement showed less unphysical refinement of rigid bodies, as well as a more accurate refinement of the phospho­rus occupancy, particularly for nanocrystalline samples. This has demonstrated the utility of PDF analysis for HA, though caution should still be observed for samples which are known to be heavily anisotropic, as this method does not allow for any representation of crystallite anisotropy. Regardless, this method presents a new approach for measuring the smallest dimension of HA crystallites.

This study presents the first examination of local order with increasing carbonate substitution in a wide range of synthetic HA samples. Phospho­rus occupancy presents a particularly useful parameter for the determination of CO_3_^2−^ concentration using real-space refinement, more accurately than can be done using Rietveld refinement. The consequence of a face-substituted (B-type) CO_3_^2−^ has been examined on a local scale, over a range of samples, and the results of real-space refinement have indicated that CO_3_^2−^ is preferentially substituted in the C1 position over the C2 position. Additionally, evidence has shown that CO_3_^2−^ affects inter­mediate (and therefore likely average) order, but not local order.

Further understanding of synthetic HA, and the methods used to characterize the local order of this material, can potentially be applied to biogenic material in the future, with implications for both biomedical and clinical settings.

## Supplementary Material

Crystal structure: contains datablock(s) global, I. DOI: 10.1107/S2053229622003400/cu3180sup1.cif

Supporting plots and table. DOI: 10.1107/S2053229622003400/cu3180sup2.pdf

CCDC reference: 2162697

## Figures and Tables

**Table 1:** Outline of sample used Sample designation is derived from the wt% CO_3_^2−^ for each sample.

Synthesis tem­per­ature	Total number of samples	Sample designations
105 °C	2	2910a, 2910b
80–90 °C	5	1.24 wt%, 4.43 wt%, 5.24 wt%, 7.52 wt%, 8.12 wt%
22 °C	13	1.96 wt%, 2.18 wt%, 2.22 wt%, 2.54 wt%, 3.40 wt%, 3.55 wt%, 4.23 wt%, 5.56 wt%, 5.83 wt%, 6.03 wt%, 6.20 wt%, 7.15 wt%, 7.98 wt%

**Table 2:** Lattice parameters for NIST SRM 640c and 2910b, giving both certified and experimental values

SRM 640c	*a* Lp (Å)	
Certified value	5.4312	
Real-space refinement	5.4423 (9)	
		
SRM 2910b	*a* Lp (Å)	*c* Lp (Å)
Certified value	9.4227	6.8886
Si-spiked sample	9.4228 (8)	6.8881 (13)
Rietveld refinement	9.4216 (6)	6.8873 (9)
Real-space refinement	9.449 (7)	6.908 (9)

**Table 3:** Results of linear regression between parameters determined through both the Rietveld refinement of Bragg data and the real-space refinement of PDF data for all samples A dash (–) signifies a relationship which was not significantly correlated (*p* < 0.05), as determined by linear regression.

Parameter	*p*	*R* ^2^ (adjusted)	Correlation
*a* Lp (Å)	0.001	0.55	0.76
*c* Lp (Å)	0.006	0.31	0.59
P *x*	0.251	0.02	–
P *y*	0.991	0.00	–
Ca1 *z*	0.528	0.00	–
Ca2 *x*	0.002	0.40	−0.65
Ca2 *y*	0.539	0.00	–
O(H) *z*	0.062	0.14	–
*z* rotation (°)	0.039	0.17	−0.46
P—O1 bond length (Å)	0.497	0.00	–
P—O2 bond length (Å)	0.936	0.00	–
P—O3 bond length (Å)	0.060	0.14	–
O1—P—O2 angle (°)	0.015	0.25	0.54
O1—P—O3 angle (°)	0.046	0.16	0.45
O1—P—O2/O3a torsion angle (°)	0.276	0.01	–
O1—P—O2/O3b torsion angle (°)	0.404	0.00	–
P occupancy	0.001	0.45	0.69
O1 occupancy	0.001	0.57	−0.77
O2 occupancy	0.001	0.47	−0.71
O3 occupancy	0.051	0.15	–
Ca1 occupancy	0.277	0.01	–
Ca2 occupancy	0.050	0.15	−0.44
O(H) occupancy	0.041	0.17	0.46

**Table 4:** Relationships between atomic fractional coordinates, rigid-body parameters, site occupancy and CO_3_^2−^ concentration A dash (–) signifies a relationship which was not significantly correlated.

	Low-tem­per­ature synthesis			High-tem­per­ature synthesis		
Parameter	*p*	*R* ^2^	Correlation	*p*	*R* ^2^	Correlation
*a* (Å)	0.005	0.53	0.73	0.321	0.32	–
*c* (Å)	0.025	0.38	0.62	0.027	0.84	0.92
P *x*	0.019	0.41	0.64	0.882	0.01	–
P *y*	0.022	0.39	0.62	0.279	0.37	–
Ca1 *z*	0.008	0.49	0.70	0.001	0.99	0.99
Ca2 *x*	0.022	0.39	0.63	0.355	0.28	–
Ca2 *y*	0.001	0.62	0.79	0.614	0.10	–
O(H) *z*	0.002	0.58	−0.76	0.013	0.090	−0.95
*z* rotation angle (°)	0.060	0.28	–	0.693	0.06	–
P—O1 bond length (Å)	0.044	0.32	0.57	0.785	0.03	–
P—O2 bond length (Å)	0.035	0.64	0.59	0.219	0.45	–
P—O3 bond length (Å)	0.006	0.51	−0.72	0.372	0.27	–
O1—P—O2 angle (°)	0.003	0.57	0.75	0.237	0.42	–
O1—P—O3 angle (°)	0.001	0.67	−0.82	0.152	0.55	–
O1—P—O2/O3a torsion angle (°)	0.049	0.31	−0.55	0.924	0.00	–
O1—P—O2/O3b torsion angle (°)	0.012	0.45	0.67	0.369	0.27	–
P occupancy	0.001	0.63	−0.79	0.004	0.95	−0.98
O1 occupancy	0.766	0.01	–	0.422	0.22	–
O2 occupancy	0.001	0.64	−0.80	0.035	0.82	−0.90
O3 occupancy	0.001	0.71	0.84	0.804	0.02	–
Ca1 occupancy	0.013	0.44	−0.67	0.308	0.33	–
Ca2 occupancy	0.008	0.25	−0.50	0.256	0.40	–
O(H) occupancy	0.026	0.38	0.61	0.134	0.58	–
