# Supplementary material for: Investigating pair distribution function use in analysis of nanocrystalline hy­droxy­apatite and carbonate-substituted hy­droxy­apatite
Source: Acta Crystallogr C Struct Chem. 2022 Apr 5;78(Pt 5):271–9. doi: 10.1107/S2053229622003400 (PMC9069248; doi:10.1107/S2053229622003400)
Supplement: Supplementary file 2 [file c-78-00271-sup2.pdf]

STRUCTURAL CHEMISTRY

Volume 78 (2022)

Supporting information for article:

Investigating pair distribution function use in analysis of nanocrystalline hydroxyapatite- and carbonate-substituted hydroxyapatite

Emily L. Arnold, Dean S. Keeble, J. P. O. Evans, Charlene Greenwood and Keith D. Rogers

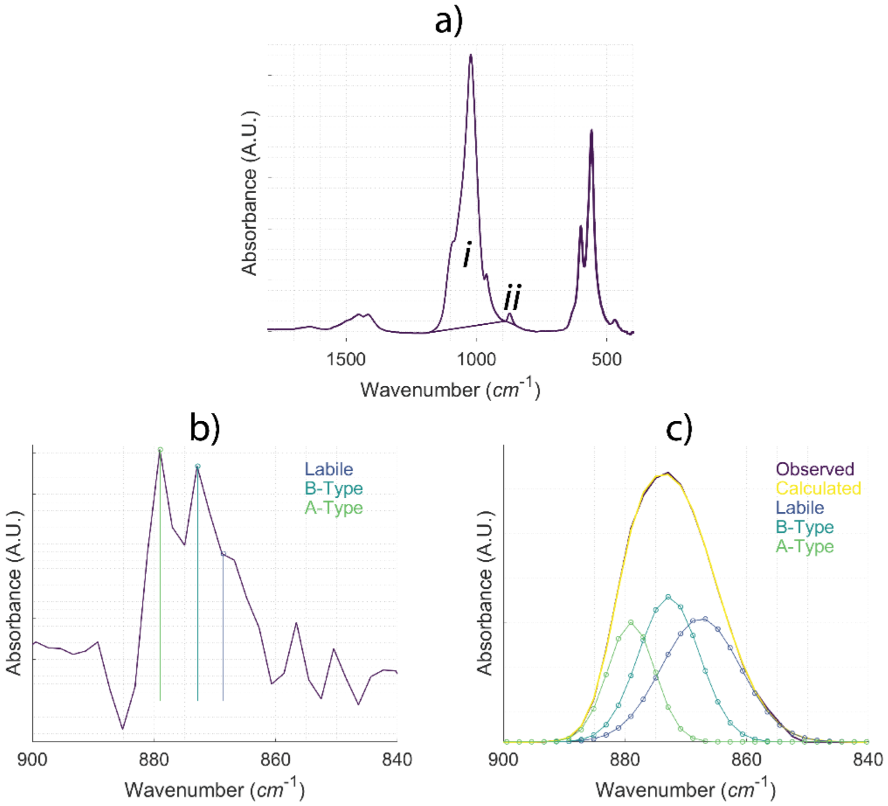

Figure S1 Example of FTIR analysis. a) shows a section of the spectrum with *i* and *ii* representing the $\nu_1\nu_3PO_4^{3-}$ and the $\nu_2CO_3^{2-}$ absorption bands respectively. b) shows the deconvoluted spectrum between 900 cm^{-1} and 840 cm^{-1}. c) shows the result of fitting three peaks to the $\nu_2CO_3^{2-}$ absorption band using PeakFit4 (Sigmaplot).

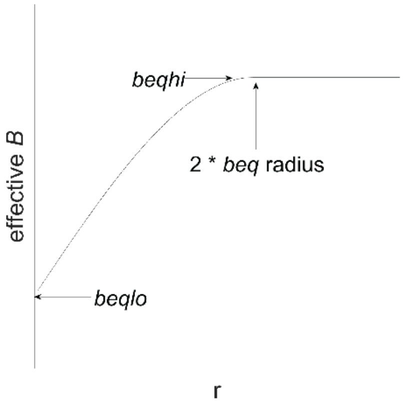

Figure S2 Effective temperature factor using spherical function.

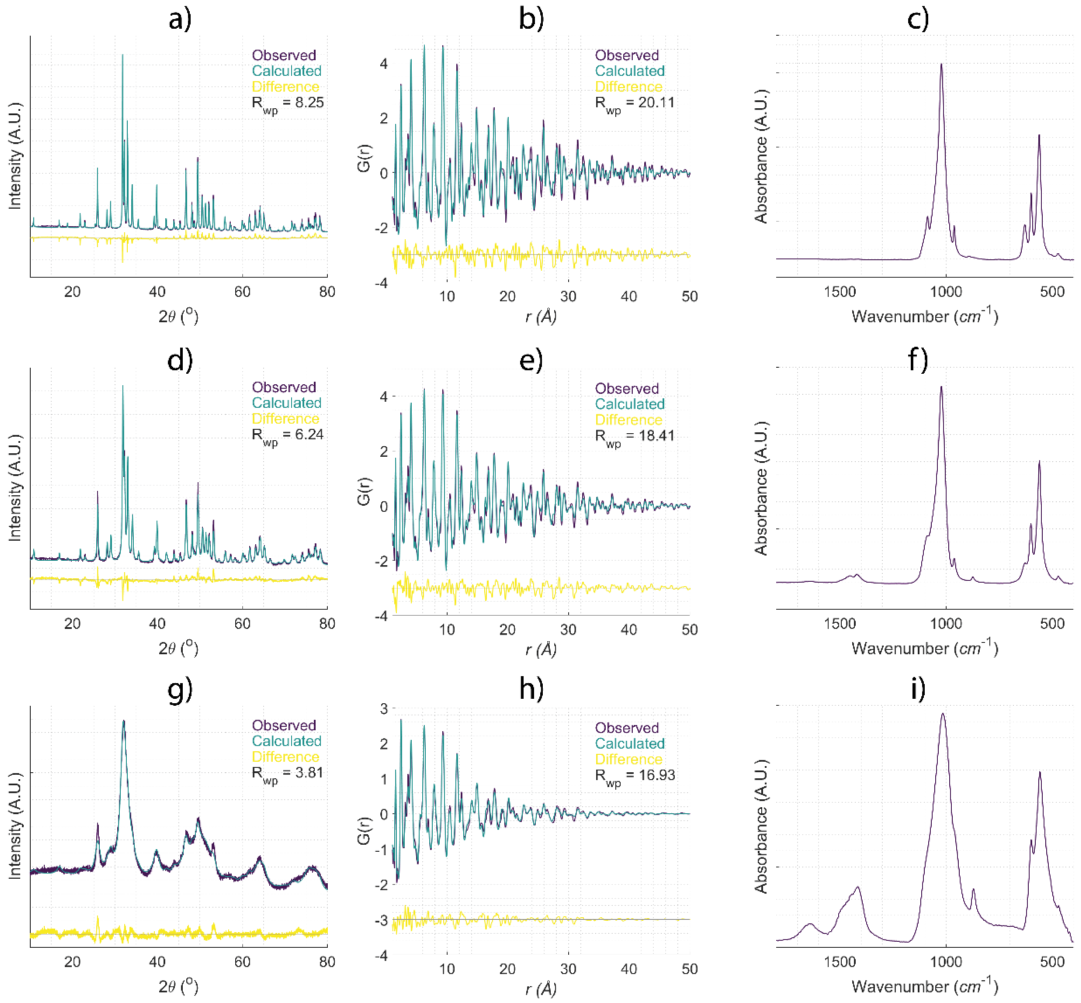

Figure S3 Examples. NIST SRM 2910b a) Rietveld refinement, b) real-space refinement and c) FTIR spectrum. High temperature (highly crystalline) synthesised 1.24 wt% d) Rietveld refinement, e) real-space refinement and f) FTIR spectrum. Low temperature (nanocrystalline) synthesised 7.98 wt% g) Rietveld refinement, h) real-space refinement and i) FTIR spectrum.

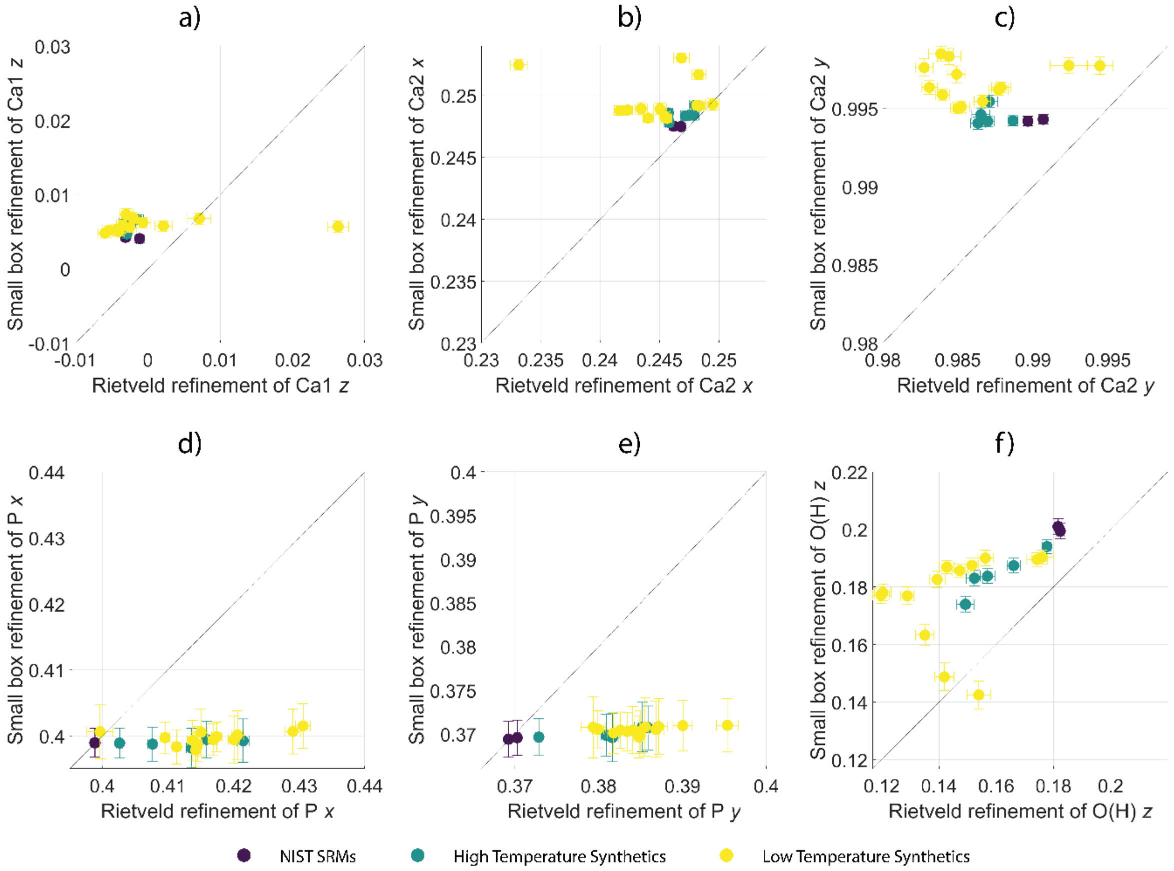

Figure S4 Comparison of fractional coordinates calculated from both Rietveld refinement of Bragg data and real-space refinement of PDF data: *a)* Ca1 *z*, *b)* Ca2 *x*, *c)* Ca2 *y*, *d)* P *x*, *e)* P *y* and *f)* O(H) *z*. Error bars represent estimated standard deviation.

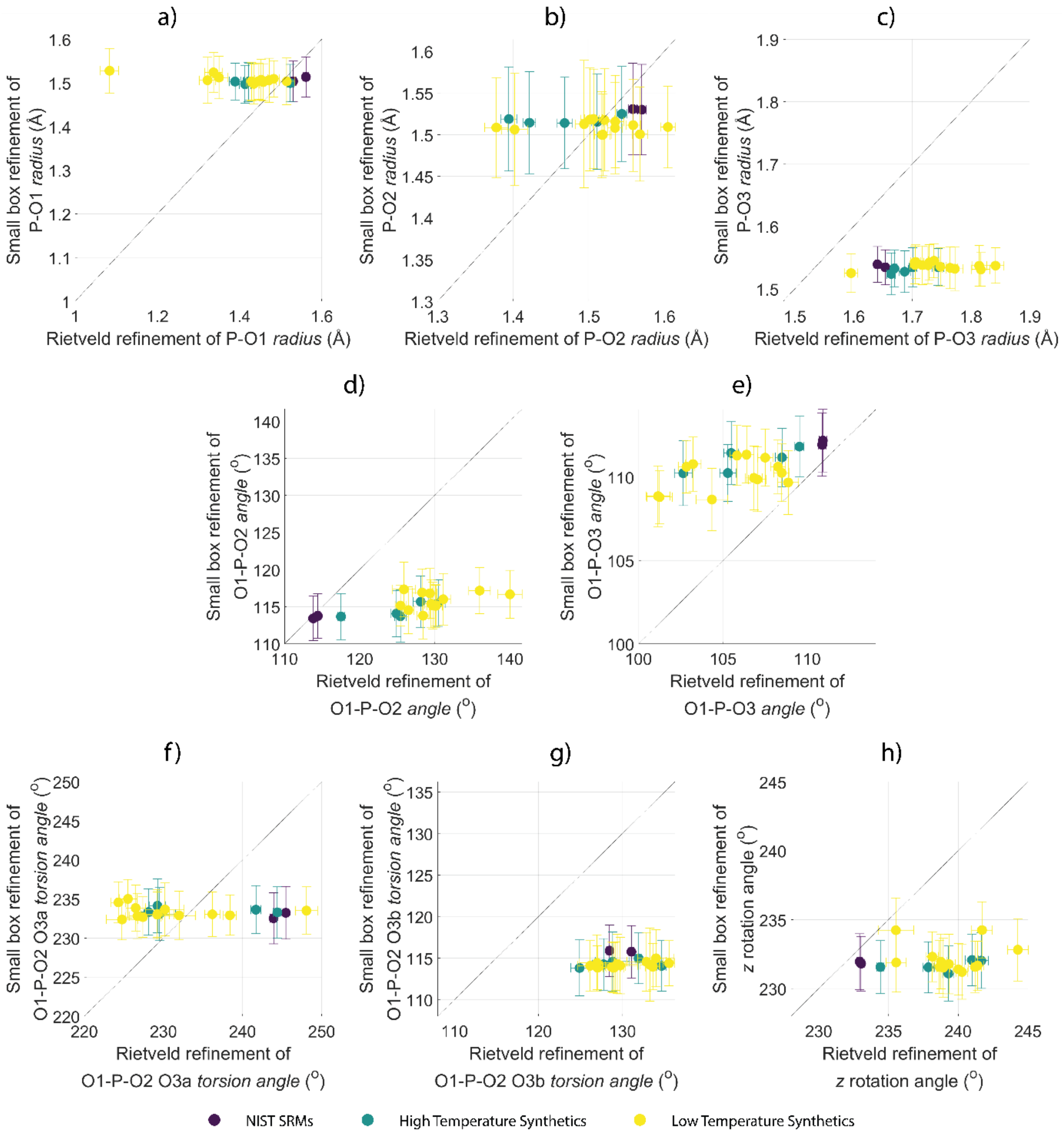

Figure S5 Comparison of rigid body parameters calculated from both Rietveld refinement of Bragg data and real-space refinement of PDF data: *a)* P-O1 bond distance, *b)* P-O2 bond distance, *c)* P-O3 bond distance, *d)* O1-P-O2 angle, *e)* O1-P-O3 angle, *f)* O1-P-O2 O3a torsion angle, *g)* O1-P-O2 O3b torsion angle and *h) z* rotation angle. Error bars represent estimated standard deviation.

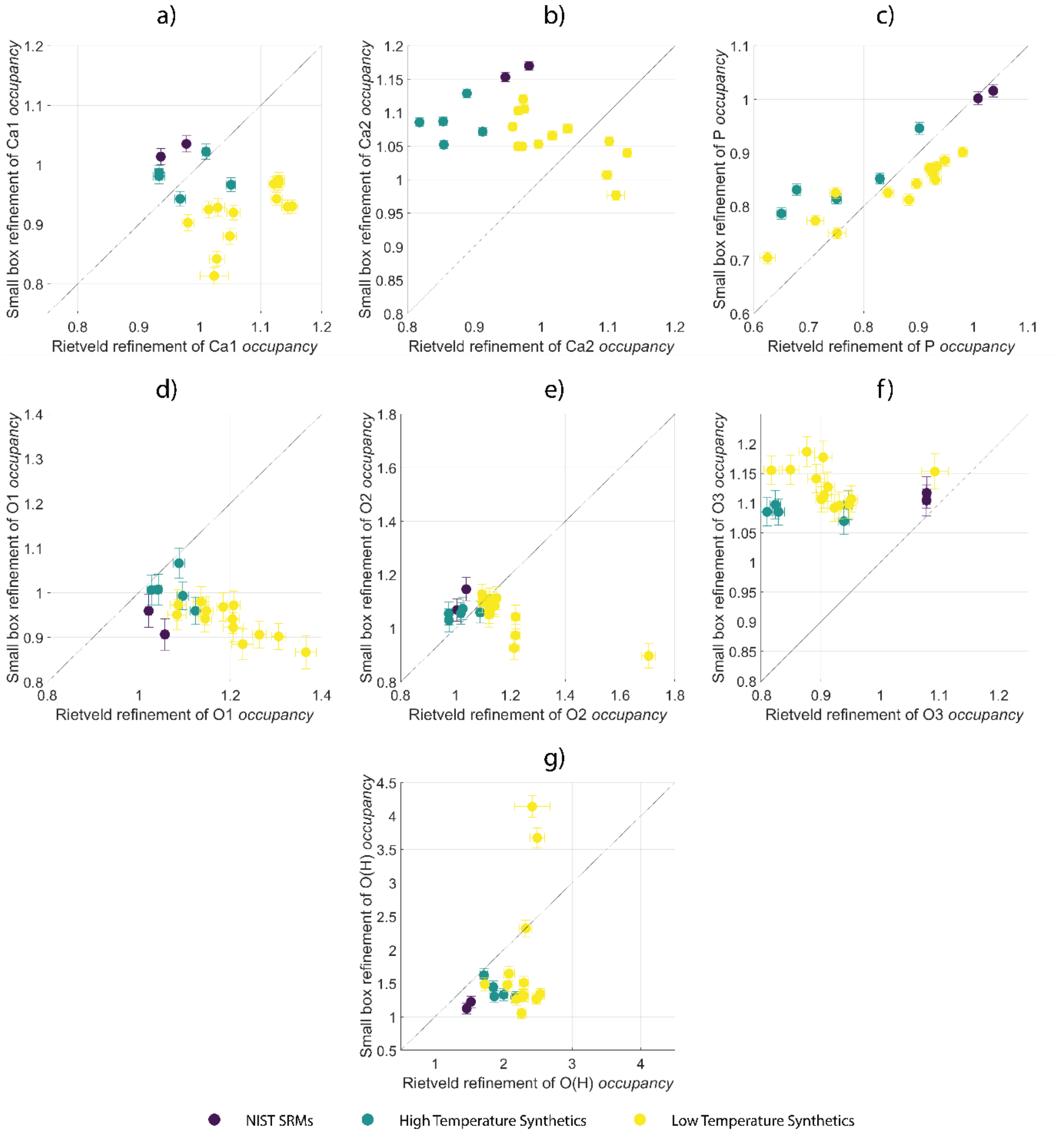

Figure S6 Comparison of occupancies calculated from both Rietveld refinement of Bragg data and real-space refinement of PDF data: *a)* Ca1 occupancy, *b)* Ca2 occupancy, *c)* P occupancy, *d)* O1 occupancy, *e)* O2 occupancy, *f)* O3 occupancy and *g)* O(H) occupancy. Error bars represent estimated standard deviation.

Table S1 Results of linear regression between expected P occupancy and the experimental P occupancy for both Rietveld and real-space refinements.

	Rietveld Refinement	Real-space Refinement
p	0.001	0.001
R^2 (adjusted)	0.62	0.72
Correlation	0.80	0.86

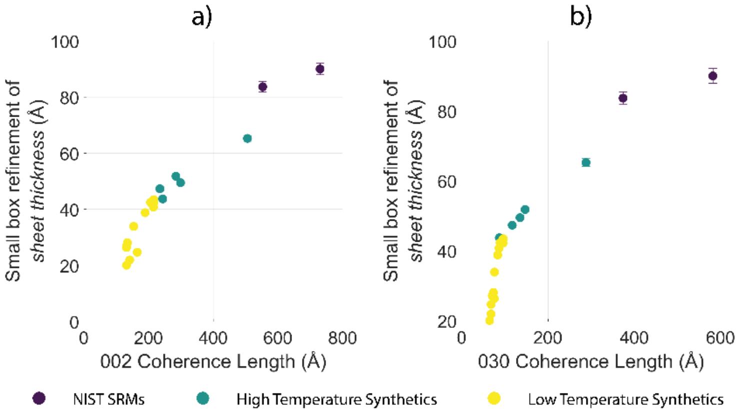

Figure S7 Comparison of sheet thickness and *a)* 002 CL and *b)* 030 CL. Error bars represent estimated standard deviation.

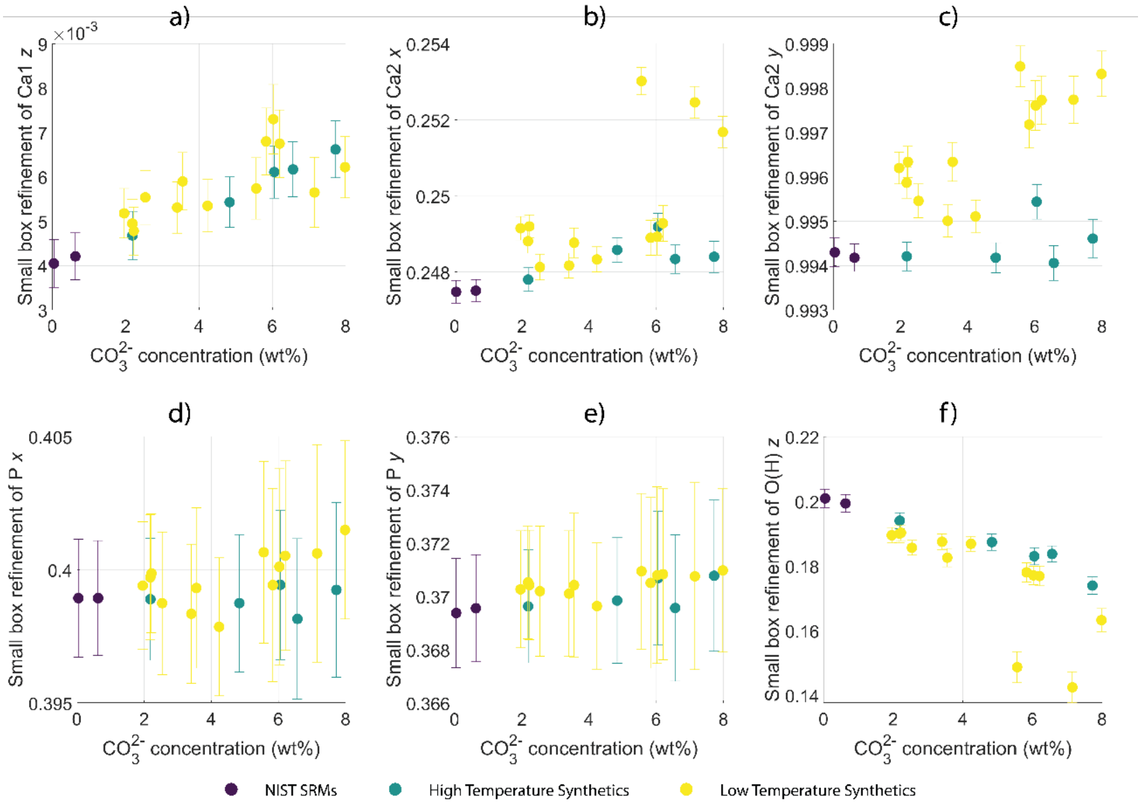

Figure S8 Relationships between atomic fractional coordinates and CO_3^{2-} concentration: *a)* Ca1 *z*, *b)* Ca2 *x*, *c)* Ca2 *y*, *d)* P *x*, *e)* P *y* and *f)* O(H) *z*. Error bars represent fitting errors. Error bars represent estimated standard deviation.

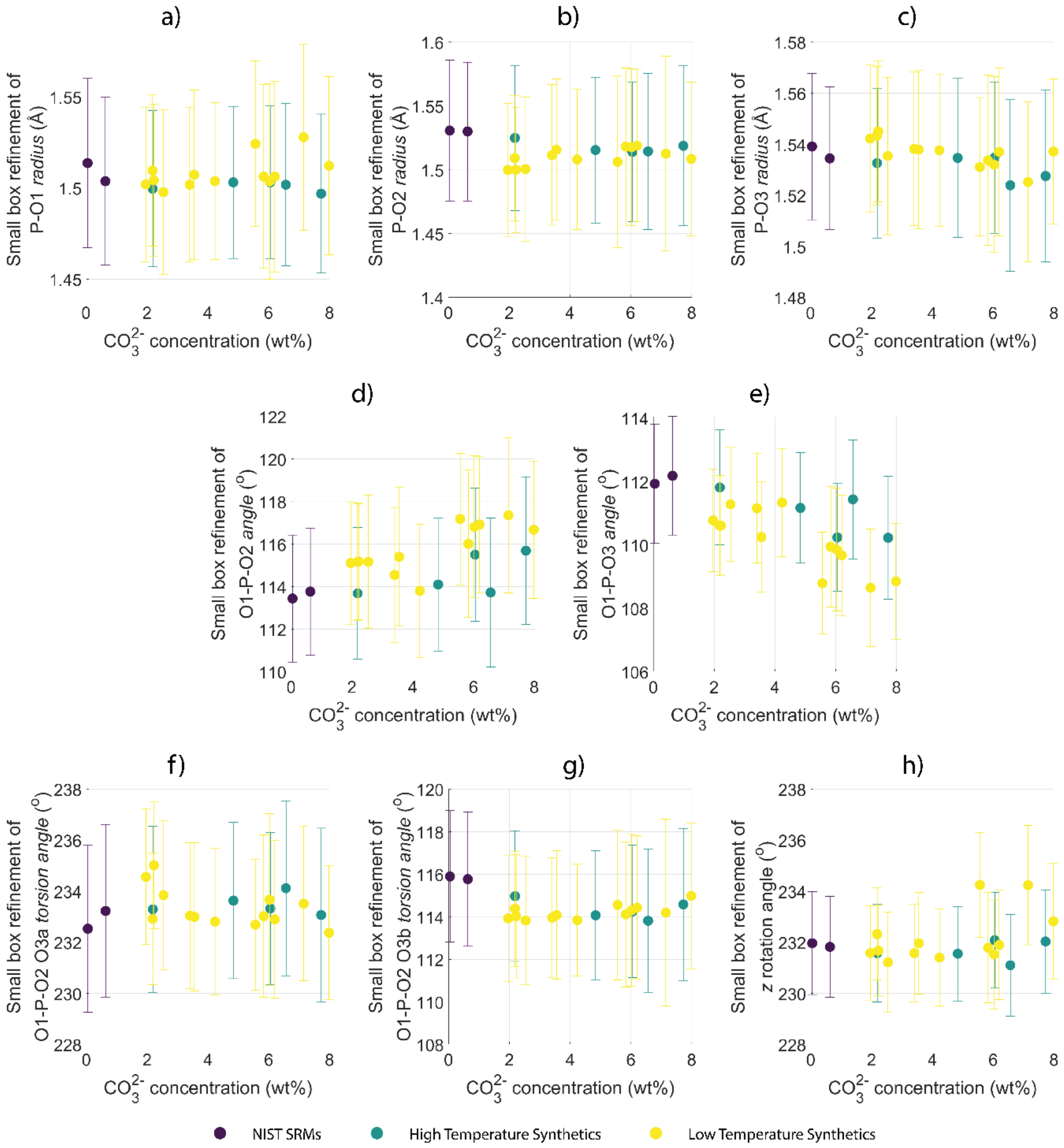

Figure S9 Relationships between rigid body parameters and CO_3^{2-} concentration: *a)* P-O1 bond distance, *b)* P-O2 bond distance, *c)* P-O3 bond distance, *d)* O1-P-O2 angle, *e)* O1-P-O3 angle, *f)* O1-P-O2 O3a torsion angle, *g)* O1-P-O2 O3b torsion angle and *h)* *z* rotation angle. Error bars represent estimated standard deviation.

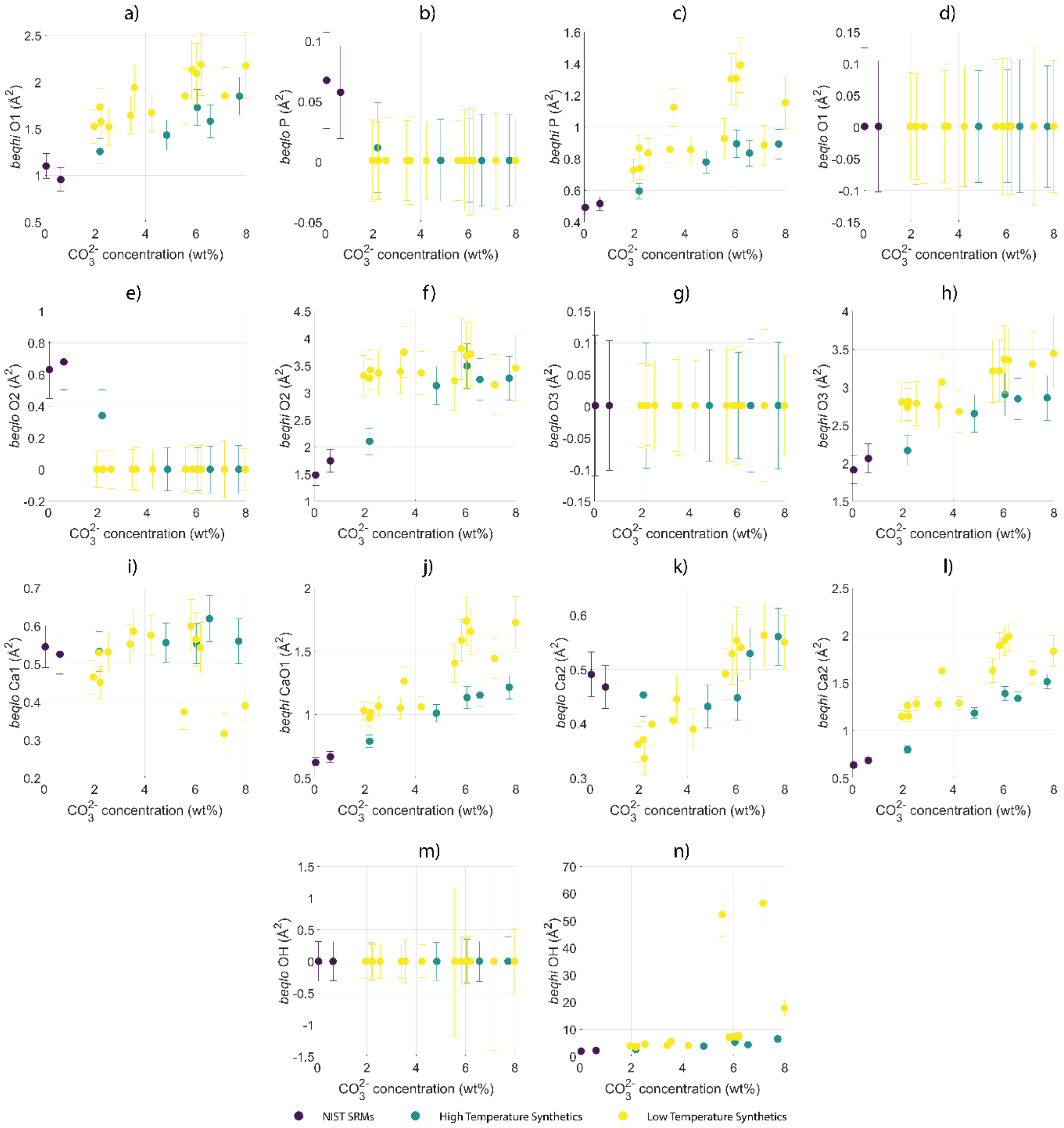

Figure S10*beq* parameters correlated to total CO_3^{2-} concentration: *a) beqlo* P, *b) beqhi* P, *c) beqlo* O1, *d) beqhi* O1, *e) beqlo* O2, *f) beqhi* O2, *g) beqlo* O3, *h) beqhi* O3, *i) beqlo* Ca1, *j) beqhi* Ca1, *k) beqlo* Ca2, *l) beqhi* Ca2, *m) beqlo* O(H) *and n) beqhi* O(H). Error bars represent estimated standard deviation.

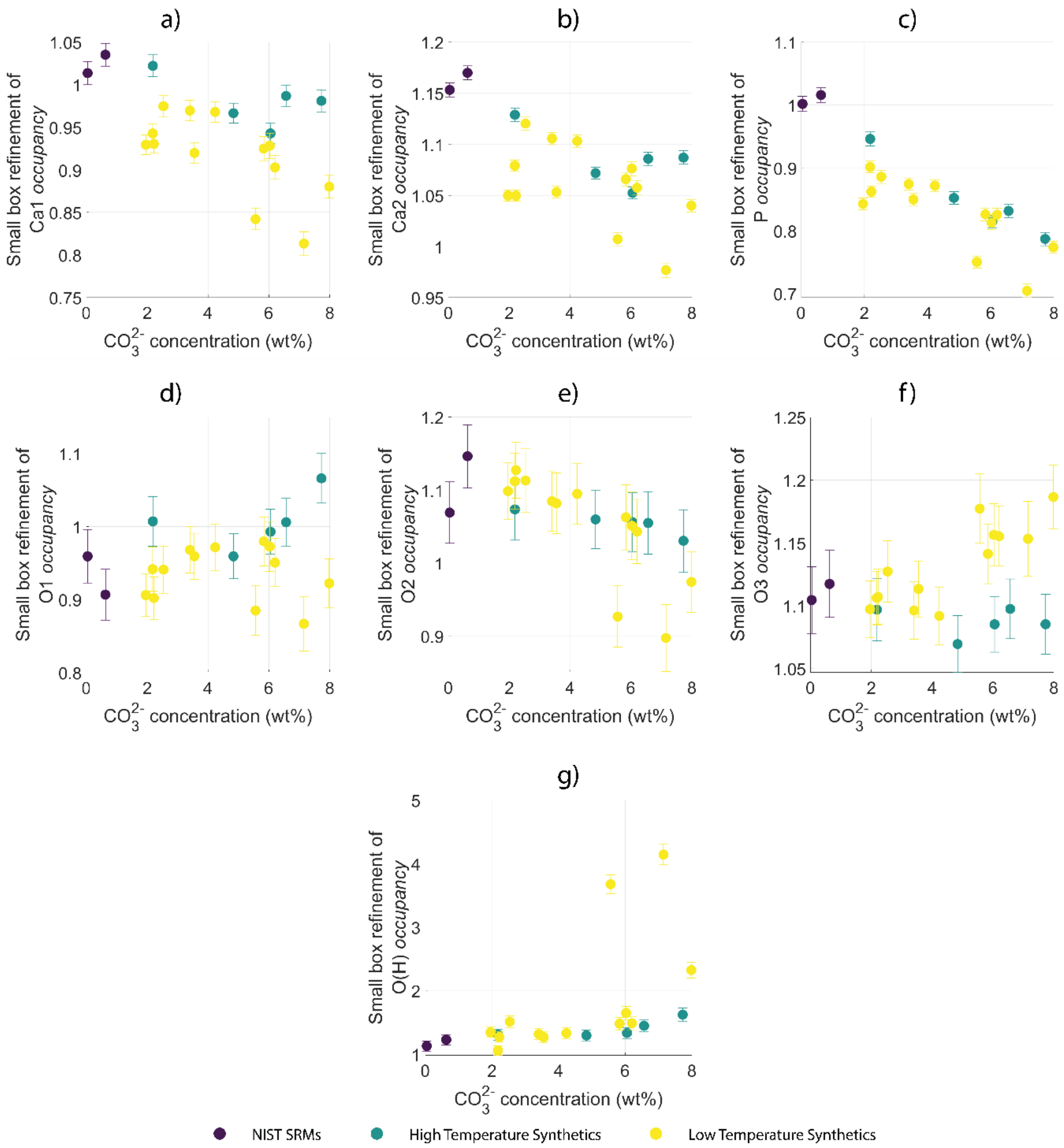

Figure S11 Relationships between occupancy and CO_3^{2-} concentration: *a)* Ca1 occupancy, *b)* Ca2 occupancy, *c)* P occupancy, *d)* O1 occupancy, *e)* O2 occupancy, *f)* O3 occupancy and *g)* O(H) occupancy. Error bars represent estimated standard deviation.
